# Whole Genome Sequencing and Multiplex qPCR Methods to Identify *Campylobacter jejuni* Encoding *cst-II* or *cst-III* Sialyltransferase

**DOI:** 10.3389/fmicb.2018.00408

**Published:** 2018-03-16

**Authors:** Jason M. Neal-McKinney, Kun C. Liu, Karen C. Jinneman, Wen-Hsin Wu, Daniel H. Rice

**Affiliations:** Pacific Northwest Laboratory, Applied Technology Center, U.S. Food and Drug Administration, Bothell, WA, United States

**Keywords:** *Campylobacter jejuni*, lipooligosaccharide (LOS), Guillain-Barré syndrome (GBS), ganglioside mimicry, whole genome sequencing, qPCR

## Abstract

*Campylobacter jejuni* causes more than 2 million cases of gastroenteritis annually in the United States, and is also linked to the autoimmune sequelae Guillan–Barre syndrome (GBS). GBS often results in flaccid paralysis, as the myelin sheaths of nerve cells are degraded by the adaptive immune response. Certain strains of *C. jejuni* modify their lipooligosaccharide (LOS) with the addition of neuraminic acid, resulting in LOS moieties that are structurally similar to gangliosides present on nerve cells. This can trigger GBS in a susceptible host, as antibodies generated against *C. jejuni* can cross-react with gangliosides, leading to demyelination of nerves and a loss of signal transduction. The goal of this study was to develop a quantitative PCR (qPCR) method and use whole genome sequencing data to detect the *Campylobacter* sialyltransferase (*cst*) genes responsible for the addition of neuraminic acid to LOS. The qPCR method was used to screen a library of 89 *C. jejuni* field samples collected by the Food and Drug Administration Pacific Northwest Lab (PNL) as well as clinical isolates transferred to PNL. *In silico* analysis was used to screen 827 *C. jejuni* genomes in the FDA GenomeTrakr SRA database. The results indicate that a majority of *C. jejuni* strains could produce LOS with ganglioside mimicry, as 43.8% of PNL isolates and 46.9% of the GenomeTrakr isolates lacked the *cst* genes. The methods described in this study can be used by public health laboratories to rapidly determine whether a *C. jejuni* isolate has the potential to induce GBS. Based on these results, a majority of *C. jejuni* in the PNL collection and submitted to GenomeTrakr have the potential to produce LOS that mimics human gangliosides.

## Introduction

*Campylobacter jejuni* is a leading bacterial cause of gastroenteritis in the United States and worldwide (Tauxe, 1992; Kaakoush et al., 2015; CDC, 2017). Infection is usually linked to the consumption of contaminated food or water, especially poultry products (Konkel et al., 2001; Sahin et al., 2015; Skarp et al., 2016). The link between *C. jejuni* and poultry is due to the fact that both wild and domestic birds can be colonized with high levels of *C. jejuni*a without causing illness to the avian host (Sahin et al., 2015; Johnson et al., 2017). Within commercial chicken flocks, *C. jejuni* spreads rapidly from bird to bird and remains present in their digestive tract until the time of slaughter (Garcia-Sanchez et al., 2017; Johnson et al., 2017). One gram of cecal contents may contain greater than 10^8^ CFU of *C. jejuni*, and can cross contaminate *C. jejuni*-free chicken carcasses during processing (Garcia-Sanchez et al., 2017; Johnson et al., 2017). As a result, raw poultry is often contaminated with *C. jejuni* at the point of sale. Cross-contamination in the kitchen can then lead to the introduction of *C. jejuni* into other food products (Sahin et al., 2015; Skarp et al., 2016).

The disease caused by *C. jejuni*, campylobacteriosis, is characterized by fever, abdominal cramps, vomiting, and diarrhea containing blood and/or fecal leukocytes (Konkel et al., 2001). Most cases are self-limiting and resolve within a few days, but antibiotics such as erythromycin may be administered in severe infections (Konkel et al., 2001). However, approximately one in a 1000 *C. jejuni* infections leads to autoimmune sequelae such as Guillan–Barre syndrome (GBS) (Tauxe, 1992; Nyati and Nyati, 2013). Campylobacteriosis is responsible for approximately 30% of all GBS cases, and a reduction in *C. jejuni* incidence can significantly reduce the burden of GBS (Tauxe, 1992; Poropatich et al., 2010; Baker et al., 2012). These autoimmune disorders are caused by the induction of an adaptive immune response that targets cells of the nervous system, and are the leading cause of paralysis in the post-polio era (Amon et al., 2012; Wijdicks and Klein, 2017). In susceptible hosts, antibodies that are generated against the surface of *C. jejuni* may also bind myelin present on the surface of nerve cells. Destruction of the myelin causes a loss of signal transduction, leading to the flaccid paralysis and loss of motor function that characterizes GBS (Nyati and Nyati, 2013; Wijdicks and Klein, 2017). It is important to note that the development of these autoimmune sequelae is dependent on both a host that is susceptible to self-recognizing antibodies, as well as the bacterial antigens that induce antibody production (Koga et al., 2006; Islam et al., 2012; Huizinga et al., 2015; Lardone et al., 2016; St Charles et al., 2017).

The lipooligosaccharide (LOS) found in the outer membrane of *C. jejuni* is the molecule responsible for inducing GBS autoantibodies (Parker et al., 2005; Koga et al., 2006; Yuki, 2007; Amon et al., 2012). The LOS of *C. jejuni* can be heavily modified via the addition of carbohydrate moieties to the O-antigen, resulting in a diverse array of LOS antigens amongst *C. jejuni* strains (Godschalk et al., 2004; Li et al., 2005; Dzieciatkowska et al., 2008; Parker et al., 2008; Semchenko et al., 2012; Stephenson et al., 2013). The addition of sialic (*n*-acetylneuraminic) acid residues to the O antigen can result in structures that are nearly identical to the GM1, GD1a, GQ1b, and GT1a gangliosides found on the surface of nerve cells (Gilbert et al., 2002; Li et al., 2005; Koga et al., 2006; Yuki, 2007). Sera taken from GBS patients have been shown to recognize one or more of these gangliosides as well as the surface of specific *C. jejuni* strains (Lardone et al., 2016). While it is not yet possible to determine who is susceptible to these autoimmune disorders, it is possible to predict whether a *C. jejuni* strain possesses sialylated LOS and has the potential to induce GBS. It is not entirely clear which particular ganglioside mimic is more likely to induce GBS, but it does appear that the addition of sialic acid (sialylation) to LOS is required for ganglioside mimicry (Godschalk et al., 2004; Koga et al., 2006; Yuki, 2007; Parker et al., 2008; Amon et al., 2012; Lardone et al., 2016). Other *Campylobacter* species, such as *Campylobacter coli*, may also produce ganglioside mimics although the mechanism and genes required are not as well-characterized (Richards et al., 2013). In this study, the genes necessary for LOS sialylation were chosen as targets for qPCR and whole genome sequencing (WGS)-based assays, so that isolates with the potential to produce ganglioside mimics could be rapidly identified.

**FIGURE 1:**
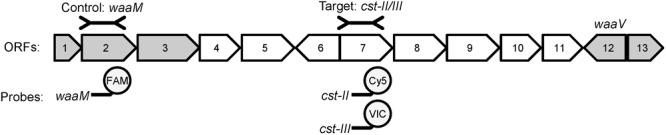
The LOS biosynthetic locus contains variable and conserved genes required for LOS gangliosdide mimicry. An example of the gene organization of LOS type A (*cst-II* positive) is shown [adapted from Parker et al. (2005)]. ORFs conserved amongst all LOS types are shown in gray, while variable ORFs are shown in white. The control and target regions used in the qPCR are shown above the diagram, and the fluorophores used for each gene are shown below. ORF2 (*waaM*) encodes an acyltransferase involved in Lipid A biosynthesis and is highly conserved. ORF7 encodes *cst-II* in LOS types A and B, and *cst-III* in LOS type C. ORF12 (*waaV*) is a highly conserved putative glycosyltransferase. The gene content of the variable region between *waaM* and *waaV* determines the LOS type.

Most of the genes responsible for LOS synthesis and modification are clustered in the genome and a typing scheme has been developed to describe the genes present (**Figure 1**) (Gilbert et al., 2002; Godschalk et al., 2004; Parker et al., 2005, 2008; Richards et al., 2013). There are more than 20 different LOS types described thus far, but LOS types A, B, and C are most commonly associated with GBS (Koga et al., 2006). These three LOS types contain a variant of the *cst* gene, which encodes a sialyltransferase required for the addition of sialic acid to LOS (Gilbert et al., 2002; Godschalk et al., 2004). LOS types A and B encode the *cst-II* gene, while type C encodes *cst-III* (Gilbert et al., 2002). The two *cst* variants can add sialic acid to a single position or multiple positions within the O-antigen of LOS to create multiple structures with mimicry to GM1, GT1a, or GD1a gangliosides (Koga et al., 2006; Yuki, 2007; Parker et al., 2008; Islam et al., 2012). In addition, interaction between LOS molecules with different sialylation patterns can produce structures that mimic additional gangliosides (Koga et al., 2015), and GBS patients can develop different anti-ganglioside antibodies in response to the same ganglioside mimics (Islam et al., 2012; Lardone et al., 2016). While it is not known which ganglioside mimic(s) are most likely to induce anti-ganglioside antibodies, it is clear that sialylation of LOS is required (Godschalk et al., 2004; Koga et al., 2006; Amon et al., 2012). Thus, the *cst* genes were chosen for use as markers for *C. jejuni* strains that can produce LOS with ganglioside mimicry. The *waaM* gene, which is required for core LOS synthesis, was used as a control as it is found in all strains regardless of LOS type (Gilbert et al., 2002). The presence of *cst-II* or *cst-III* indicates that the isolate belongs to LOS types A, B, or C, and has the potential to produce ganglioside mimics (Parker et al., 2005, 2008; Koga et al., 2006).

In this study, a multiplex qPCR method was developed to accurately detect the *cst-II* or *cst-III* genes in extracted genomic DNA. The qPCR method was then utilized to determine the prevalence of the *cst* genes in a collection of *C. jejuni* strains isolated from food and environmental samples. *In silico* analyses were also utilized to detect *cst* in *C. jejuni* genomes available through GenomeTrakr. The results of this study indicate that the majority of *C. jejuni* in GenomeTrakr and the FDA Pacific Northwest Laboratory collection have the potential to produce LOS that mimics gangliosides. The screening methods described in this study will enable food safety experts to rapidly identify isolates with sialylated LOS.

## Materials and Methods

### Bacterial Strains

All *C. jejuni* strains used are listed in Supplementary Table 1. Field strains were collected by the Food and Drug Administration Pacific Northwest Laboratory from a variety of food and environmental sources. Clinical isolates were part of an existing archival collection without any patient information. The bacteria were cultured and genomic DNA extracted as described in Liu et al. (2017). Genomic DNA (gDNA) samples used for assay controls (R4B202, R4B208, R7B122) were quantified using the ds DNA BR assay kit and a Qubit 3.0 fluorometer (Thermo Fisher Scientific, Inc.) and diluted to 500, 50, 5, and 0.5 pg/μl. All other genomic DNA samples were tested by qPCR without dilution.

### Whole Genome Sequencing

Whole genome sequencing was performed as described in Liu et al. (2016), using genomic DNA isolated from the three assay controls. Briefly, libraries were prepared using the Nextera XT kit (Illumina) and sequenced using a MiSeq instrument (Illumina) with 251 reading cycles. The paired-end reads were imported into CLC Genomics v9.5.3 and *de novo* assembly was performed using default parameters. The assembled genomic sequences were uploaded to NCBI under BioProject ID PRJNA433413. The *waaM, waaV*, and *cst-III* gene sequences from NCTC 11168 (GenBank AL111168.1) and *cst-II* sequence from RM3196 (GenBank CP012690.1) were used as references to identify homologous genes in the assembled genomes.

### Quantitative PCR

Quantitative PCR was performed using EXPRESS qPCR SuperMix Universal (Invitrogen) reagents on an ABI 7500 Fast (Applied Biosystems). The oligonucleotide primers and probes used in the study are listed in **Table 1**. All primers and the FAM and Cy5 labeled probes were produced by Integrated DNA Technologies (IDT) and the VIC labeled probe was produced by Life Technologies. The primers and probe sequences were based on the PCR strategy described in Parker et al. (2005). New primer and probe sequences for this study were designed using Primer- Basic Local Alignment Search Tool (BLAST) (Ye et al., 2012). Various primer concentrations were tested in simplex (data not shown) to determine optimal reaction conditions. The optimized multiplex oligonucleotide concentrations were: 300 mM *cstII-*F and *cstII*-R4, 200 mM *cst3*-F and *cst3*-R2, 100 mM *waaM*-F and *waaM*-R, 150 mM *orf7ab*-Cy5-BHQ2, 100 mM *cst3*-VIC-MGBNFQ, and 50 mM *waaM*-FAM-BHQ. The final reaction volume contained 15 μl 2X EXPRESS qPCR SuperMix Universal, 0.06 μl ROX dye, 1 μl of DNA template, and water and oligonucleotides to 30 μl total. The thermocycling conditions were: 2 min at 95°C, 1 cycle; 10 s at 95°C, 30 s at 60°C, 40 cycles. Sequence Detection Software v1.4 (Life Technologies) was used to analyze the data with automated baselines and thresholds. The results were plotted in Excel v14.0.7, and PCR efficiency was calculated using the tool at Thermofisher.com.

**Table 1:** Oligonucleotides used in this study.

Name	Target	Sequence (5′–3′)	Modifications^a^
*waaM*-FAM-BHQ	*waaM*	AAAAAAGGCGGTATAAGACAAATGCTAAG	5′ FAM, 3′ BHQ-1
*orf7ab*-Cy5-BHQ2	*cst-II*	TATCCCAAATGAGCATCAGGAAAATAATC	5′ Cy5, 3′ BHQ-2
*cst3*-VIC-MGBNFQ	*cst-III*	CAAAACAATTTGATGTATTTAGATGCAATCAG	5′ VIC, 3′ MGBNFQ
*waaM*-F^∗^	*waaM*	CAAATCTGTTTCCCTCAATACACTCA^∗^	None
*waaM*-R^∗^	*waaM*	TTTAATCTTACGCTTTCGTTTTCTAC^∗^	None
*cstII*-F^∗^	*cst-II*	ACTACACTTTAAAACATTTAATCCAAAATCA^∗^	None
*cstII*-R4	*cst-II*	AAGATAAATTTCTTTGTATCCTAGGG	None
*cst3*-F	*cst-III*	ATGCTTTGGTATGCGGTAATGG	None
*cst3*-R2	*cst-III*	TCCACTAGTAATTCTTTGCCTATTG	None

*^∗^Primer sequence from Parker et al. (2005). ^*a*^BHQ, Black Hole Quencher; MGBNFQ, Minor groove binding non-fluorescent quencher.*

### SRA Assembly and BLAST

The SRA files for the GenomeTrakr *Campylobacter* Umbrella Project (PRJNA258021) were downloaded on 04/25/2017 and imported as paired-end Illumina reads into CLC Genomics v9.5.3. *De novo* assembly was performed using a workflow consisting of adapter (Nextera adapter sequences) and quality trimming, followed by *de novo* assembly using default parameters. The BLAST function in CLC Genomics was used to identify contigs that contained *waaM, waaV, cst-II*, and *cst-III* using the NCTC11168 and RM3196 reference sequences. Only BLAST hits with an e-value of 0 were used for downstream analysis. The contig lists generated by the BLAST search were imported into Excel v14.0.7 and compared to identify contigs containing multiple genes of interest.

## Results and Discussion

**FIGURE 2:**
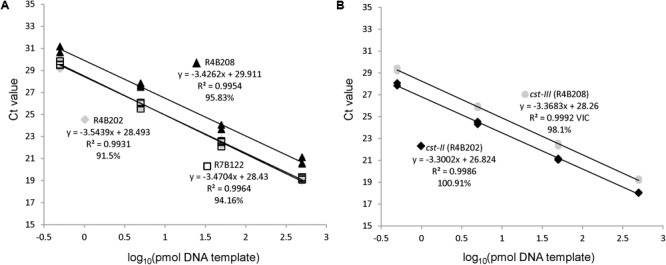
The qPCR assay efficiently amplifies *cst-II, cst-III*, and *waaM*. Serial 10-fold dilutions of control strains R4B202 (*cst-II*), R4B208 (*cst-III*), and R7B122 (*cst-*negative) were used as template in the multiplex qPCR assay to determine the PCR efficiency of the *waaM* **(A)** and *cst-II/cst-III* **(B)** targets.

A rapid qPCR method was developed to detect the *cst-II* and *cst-III* genes based on previous work demonstrating that these genes are required for the addition of sialic acid to *Campylobacter* LOS (Gilbert et al., 2002; Godschalk et al., 2004; Yuki, 2007; Parker et al., 2008). Primers specific to the *cst* genes were adapted from the typing strategy described by Parker et al. (2005) (**Table 1**). Probes within the amplicons were designed using PrimerBLAST, such that they recognize all *cst*-*II, cst-III*, and *waaM* sequences in the National Center for Biotechnology Information (NCBI) database. Primer and probe concentrations were adjusted for maximal detection sensitivity of the *cst* genes. A limiting concentration of primers was used for the *waaM* control gene, so that amplification would not interfere with detection of the *cst* targets. The *C. jejuni* strains used for testing the specificity of the qPCR assay are listed in Supplementary Table 1. The LOS gene content in each control strain (R4-B2-02, R4-B2-08, and R7-B1-22) was confirmed by WGS. All qPCR reactions yielded the result expected based on the genomic sequences of the *C. jejuni* strains. The qPCR method was tested using dilutions of gDNA from the three control strains. DNA quantities of 500, 50, 5, and 0.5 pg were used as template to determine the sensitivity and efficiency of the qPCR amplification (**Figure 2**). The amplification efficiency for all three targets was greater than 95% across the four-log magnitude concentration range. Importantly, detection of the *cst-II* and *cst-III* genes was more sensitive than detection of the *waaM* gene used as a control. These results indicate that the qPCR assay is specific across a wide range of DNA concentrations, and that the *waaM* control is appropriate.

The qPCR assay was then used to screen 89 *C. jejuni* strains in an archive at Food and Drug Administration Pacific Northwest Laboratory (FDA-PNL). These strains were isolated from field (i.e., food and environmental sources) and clinical samples, and collected over a 20-year period (Supplementary Table 1). Using the qPCR assay, *cst-II* was found in 36 samples (40.5%), *cst-III* was found in 13 samples (14.6%), and *waaM* was found in all samples tested (**Table 2**). Interestingly, the 38 clinical samples had more *cst*-negative isolates (57.9%) than the 51 field samples (33.3%). The qPCR screening method could be employed in future studies to determine the prevalence of *cst-II* and *cst-III* isolates in other geographic regions or environmental sources.

**Table 2:** Multiplex qPCR screen of 89 *C. jejuni* isolates.

Target	# Positive	Percent
**All isolates**		
*waaM*	89	100
*cst-II*	36	40.5
*cst-III*	13	14.6
Neg.^a^	39	43.8
**Field isolates**		
*waaM*	51	100
*cst-II*	26	51.0
*cst-III*	7	13.7
Neg.	17	33.3
**Clinical isolates**		
*waaM*	38	100
*cst-II*	10	26.3
*cst-III*	6	15.8
Neg.	22	57.9

*^*a*^Neg., no *cst* genes detected.*

The multiplex qPCR presented in this study has several major differences compared to sequencing or PCR based approaches previously described (Gilbert et al., 2002; Parker et al., 2005, 2008). Our multiplex qPCR method is faster to perform than Sanger sequencing or next generation sequencing, which require extensive time for library preparation, sequencing, and data analysis. The qPCR assay is also multiplexed and uses specific fluorescent probes for detection, so the reaction can be performed and analyzed using a single instrument in less than an hour of runtime. However, the qPCR assay presented here only determines if a strain possesses one of the two sialyltransferase genes; determination of the LOS class type or *cst-II* polymorphisms would require additional information. Based on the requirement of *cst-II* or *cst-III* for sialylation of LOS (Gilbert et al., 2002; Godschalk et al., 2004), we believe that our qPCR assay provides a simplified alternative method for the detection of *C. jejuni* with the potential to produce sialylated LOS. WGS could then be performed to fully characterize the LOS locus in isolates of interest.

**FIGURE 3:**
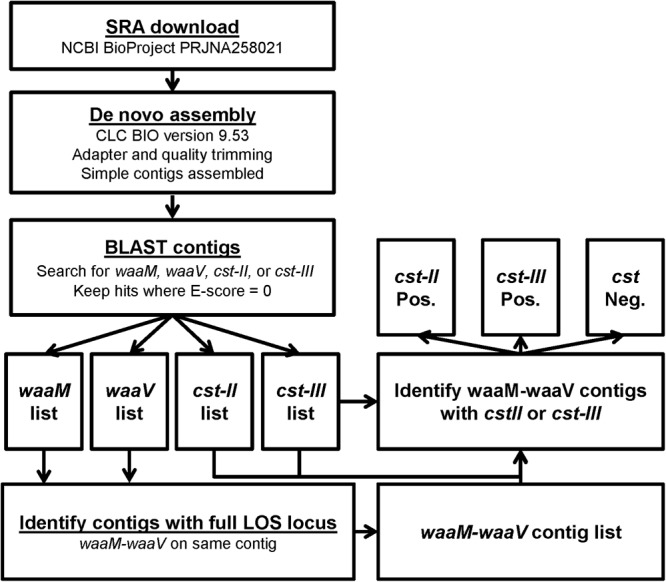
Workflow for the determination of *cst*-type from GenomeTrakr SRA data. The strategy for identifying *cst* genes from SRA files starts with *de novo* assembly, followed by BLAST searches for the four genes of interest. To ensure that the variable genes of the LOS locus were present in an SRA, contigs were identified that contained both flanking genes, *waaM* and *waaV*. The *waaM-waaV* contigs were then determined to be *cst-II* positive or *cst-III* positive if the gene was found in the same contig, and *cst* negative if the genes were not detected.

An *in silico* approach was used to screen 827 *C. jejuni* genomes sequenced as part of the GenomeTrakr project, available from NCBI BioProject PRJNA258021. Fortunately, the genes involved in LOS sialylation are contained within a defined LOS biosynthetic locus flanked by the *waaV* and *waaM* genes, which are conserved among *C. jejuni* strains. The genome of *C. jejuni*-type strain NCTC 11168 was used to obtain reference sequences for *waaM, waaV*, and *cst-III*, while strain 81-176 was used for *cst-II*. Using the BLAST, sequences were identified within the RefSeq genome database that contained both *waaM* and *waaV*, indicating that the complete LOS locus was represented in the available sequence. BLAST searches were then performed to identify genomic sequences containing *cst-II* or *cst-III* (**Figure 3**). In total, 405 complete LOS loci were identified, with 177 (43.7%) containing *cst-II*, 38 (9.4%) containing *cst-III*, and 190 (46.9%) that were negative for either *cst* gene (**Tables 3A,B**). For the 347 food isolates and 33 environmental isolates, 49.0 and 48.5% were *cst-*negative, respectively. In contrast, only 16% of the 25 clinical isolates were *cst*-negative. For the 422 genomic sequences without a complete LOS locus (**Table 3C**), 123 (29.1%) contained *cst-II* and 118 (28.0%) contained *cst-III.* No genome assemblies were found to contain both *cst-II* and *cst-III*. It is important to note that more than half sequences in the GenomeTrakr databases did not have enough coverage to complete the LOS locus, which ranges in size from ∼7.8 kb (LOS class F) to 15.2 kb (LOS class E). This is possibly due to the low GC content or homopolymeric tracts in this region which can affect sequencing. Although this database is not a fully accurate representation of *C. jejuni* strain diversity in nature, it is interesting to note that greater than 50% of strains collected from food production areas and clinical samples have the potential to produce ganglioside mimics.

**Table 3:** *In silico* analysis of 829 GenomeTrakr Sequence Read Archives (SRAs) from NCBI BioProject PRJNA258021.

All GenomeTrakr Isolates	Total		Percent	
**(A)**				
SRAs	827		100	
*waaV* sequences	816		98.7	
*waaM* sequences	800		96.7	
*cstII* sequences	300		36.3	
*cstIII* sequences	156		18.9	
Contiguous *waam-waaV*	405		49.0	
Non-contiguous *waaM-waaV*	422		51.0	
**(B)**				
	**Total**	**Food**	**Enviro.**	**Clinical**

Contiguous waam-waaV	405 (100%)	347 (85.7%)	33 (8.1%)	25 (6.2%)
*cstII-*positive	177 (43.7%)	148 (42.7%)	16 (48.5%)	13 (52%)
*cstIII*-positive	38 (9.4%)	29 (8.4%)	1 (3%)	8 (32%)
*cst-*negative	190 (46.9%)	170 (49.0%)	16 (48.5%)	4 (16%)
**(C)**				
	**Total**		**Percent**	

Non-contiguous waaM-waaV	422		100	
*cstII-*positive	123		29.1	
*cstIII*-positive	118		28.0	
*cst-negative*	181		42.9	

The strains present in the GenomeTrakr database were collected between 2002 and 2017, with 738 isolates from the United States and 89 isolates from the United Kingdom. All isolates from the United Kingdom were collected from retail meat samples by Fera and sequenced as part of a joint project with the U.S. Food and Drug Administration. In the U.S., food, environmental, and clinical samples were obtained from 12 states (AK, CA, CT, GA, IA, MD, MN, NY, OH, OR, TN, and TX). The dates and sources of isolates uploaded to GenomeTrakr vary widely between contributing labs; for example, all 25 samples from AK were collected from environmental sources between 2009 and2013, whereas all 115 samples from CT were collected from poultry meat between 2002 and2003. Additionally, some genomic sequences may be from identical or highly related isolates collected from the same source. Given these differences in sample collection, it is difficult to assess whether strains that possess *cst-II* or *cst-III* are linked to certain sources or geographical locations. However, the GenomeTrakr database is the most representative database of U.S. *C. jejuni* isolates available, and will continue to grow in size and usefulness as newly isolated sequences are added.

The goal of this study was to examine the prevalence of *C. jejuni* with the potential for LOS sialylation in the food supply. Interestingly, the qPCR assay and the *in silico* analysis of WGSs yielded similar numbers of *cst-*positive results. By qPCR 40.5 and 14.6% of isolates were positive for *cst-II* and *cst-III*, respectively, compared to 43.7 and 9.4%, respectively, for the *in silico* analysis. The percentage of *cst-*positive isolates is also consistent with previous studies indicating that roughly 50% of isolates contain a *cst* gene (Hardy et al., 2011; Amon et al., 2012; Ellstrom et al., 2013, 2016; Duarte et al., 2016).

While the qPCR assay differentiates between *cst-II* and *cst-III* variants, it does not differentiate between *cst-II* with Asn51 or Thr51. The *cst-III* and *cst-II* Thr51 genes encode for a sialyltransferase with α-2-3 activity, while *cst-II* Asn51 encodes a bi-functional sialyltransferase with α-2-3 and α-2-8 activity (Koga et al., 2006). As a result, *cst-II* Asn51 isolates can also produce GT1a-like and GQ1b-like LOS as well as GM1-like and GD1a-like LOS (Hardy et al., 2011). In this study, it was found that of the 300 *cst-II* sequences in the *Campylobacter* GenomeTrakr BioProject (PRJNA258021), 230 (76.7%) had Asn51, while 70 (23.3%) had Thr51.

This indicates that the majority of *cst-II* positive isolates have the ability to produce GT1a/GQ1b-like LOS and could potentially result in a different repertoire of anti-ganglioside antibodies. This could be an important distinction, as the clinical symptoms of GBS are influenced by the ganglioside specificity of the autoantibodies. Specifically, Miller Fisher syndrome, a variant of GBS, is thought to be caused by anti-GQ1b antibodies (Susuki et al., 2001; Yuki, 2007).

Further complicating the link between *cst* genotype and human disease is the fact that a given isolate of *C. jejuni* often contains multiple LOS structures (Godschalk et al., 2004; Li et al., 2005; Koga et al., 2006; Dzieciatkowska et al., 2008). Some LOS molecules will be unmodified by *cst*, while other LOS molecules may have single or multiple sialic acid groups, resulting in a mixture of GD1a, GM1, GT1a, and GQ1b epitopes on the surface of the outermembrane. In addition, it has been proposed that combinations of different LOS epitopes with ganglioside mimicry could stimulate the production of autoantibodies with specificity against additional gangliosides (Li et al., 2005; Koga et al., 2015). Additional research will need to be performed to determine the LOS structures present on *C. jejuni* with *cst-III, cst-II* Asn51 or *cst-II* Thr51, as well as the host immune response to the different ganglioside mimics.

In summary, a qPCR protocol was developed and WGS data available on NCBI analyzed to determine the *cst* genotype of *C. jejuni* strains. Using these methods, it was determined that nearly 60% of *C. jejuni* isolates contain the *cst* genes required to modify LOS and produce ganglioside mimics. In addition, ∼75% of *cst-II* isolates have Asn51 and are capable of stimulating additional antibody specificities. Taken together, these results indicate that approximately half of *C. jejuni* strains have the potential to produce four different ganglioside mimics. Additional studies will need to be performed to determine the role of gene expression and the quantities of each LOS structure present in the outermembrane. In the future, the qPCR assay or WGS could be used to rapidly determine whether a food or outbreak isolate has the potential to produce ganglioside mimics.

## Author Contributions

JN-M and KL: bacterial isolates were grown, DNA was extracted, and data analysis was performed. KL and KJ: whole genome sequencing was performed. JN-M: completed the qPCR experiments, as well as SRA assembly and analysis. JN-M, KL, KJ, W-HW, and DR contributed to the experimental design and review of the manuscript.

## Disclaimer

The views presented in this work do not necessarily reflect those ofthe U.S. Food and Drug Administration nor do the authors specifically endorse the listed instrumentation or products.

## Conflict of Interest Statement

The authors declare that the research was conducted in the absence of any commercial or financial relationships that could be construed as a potential conflict of interest.
