# Supplementary material for: Whole Genome Sequencing and Multiplex qPCR Methods to Identify Campylobacter jejuni Encoding cst-II or cst-III Sialyltransferase
Source: Front Microbiol. 2018 Mar 16;9:408. doi: 10.3389/fmicb.2018.00408 (PMC5865068; doi:10.3389/fmicb.2018.00408)
Supplement: Supplementary file 1 [file Table_1.DOCX]

**Supplemental Table 1. Results of qPCR assay for 89 *C. jejuni* isolates from FDA-PNL**

| **Isolate** | ***waaM* Mean Ct^a^** | ***cst-II* Mean Ct** | ***cst-III* Mean Ct** | ***cst* Type** | **Source** |
| --- | --- | --- | --- | --- | --- |
| R4-B2-02* | 14.50 | 13.63 | ND^b^ | *cstII* | Field isolate |
| R4-B2-05 | 14.43 | 13.32 | 23.97 | *cstII* | Field isolate |
| R4-B2-07 | 13.62 | 13.65 | ND | *cstII* | Field isolate |
| R4-B2-08* | 14.25 | ND | 13.83 | *cstIII* | Field isolate |
| R4-B2-09 | 13.45 | ND | ND | Neg^c^ | Field isolate |
| R4-B2-14 | 14.76 | 13.67 | ND | *cstII* | Field isolate |
| R4-B2-27 | 14.52 | 14.68 | ND | *cstII* | Field isolate |
| R4-B2-28 | 14.88 | ND | 13.95 | *cstIII* | Field isolate |
| R4-B2-34 | 13.67 | 13.33 | 14.35 | *cstII cstIII* | Field isolate |
| R4-B2-38 | 15.19 | 15.02 | ND | *cstII* | Field isolate |
| R4-B2-42 | 16.87 | ND | 15.82 | *cstIII* | Field isolate |
| R4-B4-09 | 14.40 | 13.66 | ND | *cstII* | Field isolate |
| R4-B4-10 | 15.00 | 14.55 | ND | *cstII* | Field isolate |
| R4-B4-11 | 16.30 | 14.69 | ND | *cstII* | Field isolate |
| R4-B4-13 | 15.98 | ND | ND | Neg | Field isolate |
| R4-B4-15 | 14.36 | ND | ND | Neg | Field isolate |
| R4-B4-19 | 14.70 | ND | ND | Neg | Field isolate |
| R4-B4-20 | 15.40 | 14.49 | ND | *cstII* | Field isolate |
| R4-B4-25 | 14.76 | 14.13 | ND | *cstII* | Field isolate |
| R4-B4-26 | 14.53 | ND | ND | Neg | Field isolate |
| R4-B4-31 | 13.90 | 13.05 | ND | *cstII* | Field isolate |
| R4-B4-32 | 14.44 | ND | ND | Neg | Field isolate |
| R4-B4-37 | 13.71 | ND | ND | Neg | Field isolate |
| R4-B4-38 | 13.51 | 13.53 | ND | *cstII* | Field isolate |
| R4-B4-43 | 14.82 | 14.44 | 25.15 | *cstII* | Field isolate |
| R4-B4-44 | 14.08 | ND | ND | Neg | Field isolate |
| R4-B4-49 | 13.25 | ND | ND | Neg | Field isolate |
| R7-B1-01 | 15.67 | ND | 13.96 | *cstIII* | Field isolate |
| R7-B1-06 | 14.97 | 15.80 | ND | *cstII* | Field isolate |
| R7-B1-10 | 39.57 | 15.11 | ND | *cstII* | Field isolate |
| R7-B1-14 | 12.95 | 12.85 | ND | *cstII* | Field isolate |
| R7-B1-16 | 12.42 | 13.77 | ND | *cstII* | Field isolate |
| R7-B1-18 | ND | 13.75 | ND | *cstII* | Field isolate |
| R7-B1-22* | 12.49 | ND | ND | Neg | Field isolate |
| R7-B1-24 | 14.45 | 13.55 | ND | *cstII* | Field isolate |
| R7-B1-26 | 11.46 | 15.35 | ND | *cstII* | Field isolate |
| R7-B1-28 | 13.64 | 14.64 | ND | *cstII* | Field isolate |
| R7-B1-33 | 15.33 | 13.79 | 34.38 | *cstII* | Field isolate |
| R7-B1-37 | 14.94 | ND | 15.38 | *cstIII* | Field isolate |
| R7-B1-42 | ND | 13.95 | ND | *cstII* | Field isolate |
| R7-B1-46 | 13.83 | ND | 13.41 | *cstIII* | Field isolate |
| R7-B1-48 | 13.86 | 14.24 | 23.99 | *cstII* | Clinical |
| R7-B1-70 | 15.74 | 15.32 | ND | *cstII* | Field isolate |
| R7-B2-01 | 13.05 | 13.34 | ND | *cstII* | Field isolate |
| R7-B2-34 | 16.29 | ND | ND | Neg | Clinical |
| R7-B2-55 | 18.72 | ND | 20.83 | *cstIII* | Field isolate |
| R7-B4-42 | 32.14 | ND | ND | Neg | Field isolate |
| R7-B5-53 | 19.27 | ND | ND | Neg | Field isolate |
| R7-B5-57 | 16.84 | ND | ND | Neg | Field isolate |
| R7-B5-61 | 16.44 | ND | ND | Neg | Field isolate |
| R7-B5-75 | 15.21 | ND | ND | Neg | Field isolate |
| R7-B5-86 | 14.93 | ND | ND | Neg | Field isolate |
| R7-B5-87 | 14.60 | ND | ND | Neg | Field isolate |
| 20A-01 | 14.69 | 13.07 | ND | *cstII* | Clinical |
| 20A-05 | 15.13 | ND | ND | Neg | Clinical |
| 20A-06 | 11.69 | ND | 19.37 | *cstIII* | Clinical |
| 20A-08 | 15.38 | 12.36 | ND | *cstII* | Clinical |
| 20A-10 | 15.50 | ND | ND | Neg | Clinical |
| 20A-14 | 14.14 | ND | ND | Neg | Clinical |
| 20A-16 | ND | 12.19 | ND | *cstII* | Clinical |
| 20A-17 | 16.68 | ND | ND | Neg | Clinical |
| 20A-18 | 14.80 | ND | ND | Neg | Clinical |
| 20A-21 | 16.91 | ND | ND | Neg | Clinical |
| 20A-24 | 21.02 | ND | 20.25 | *cstIII* | Clinical |
| 20A-29 | 17.97 | 12.82 | ND | *cstII* | Clinical |
| 20A-31 | 15.96 | ND | ND | Neg | Clinical |
| 20A-33 | 14.57 | ND | ND | Neg | Clinical |
| 20A-34 | 19.24 | ND | ND | Neg | Clinical |
| 20A-36 | 19.37 | 14.27 | ND | *cstII* | Clinical |
| 20A-40 | 24.47 | 12.83 | ND | *cstII* | Clinical |
| 20A-41 | 14.27 | ND | ND | Neg | Clinical |
| 20A-43 | 13.98 | ND | ND | Neg | Clinical |
| 20A-44 | 15.38 | ND | ND | Neg | Clinical |
| 20A-48 | 17.57 | ND | ND | Neg | Clinical |
| 20A-49 | 14.53 | ND | ND | Neg | Clinical |
| 20A-50 | 18.59 | ND | 20.09 | *cstIII* | Clinical |
| 20A-53 | 32.53 | 33.51 | ND | *cstII* | Clinical |
| 20A-54 | 16.77 | ND | ND | Neg | Clinical |
| 20A-57 | 13.77 | ND | ND | Neg | Clinical |
| 20A-58 | 17.00 | ND | ND | Neg | Clinical |
| 20A-59 | 12.86 | ND | 19.58 | *cstIII* | Clinical |
| 20A-61 | 14.30 | ND | ND | Neg | Clinical |
| 20A-65 | 16.92 | 12.20 | ND | *cstII* | Clinical |
| 20A-66 | 31.42 | 35.53 | ND | *cstII* | Clinical |
| 20A-67 | 16.66 | ND | ND | Neg | Clinical |
| 20A-69 | 18.09 | ND | 20.35 | *cstIII* | Clinical |
| 20A-75 | 15.09 | ND | ND | Neg | Clinical |
| 20A-78 | 12.95 | ND | ND | Neg | Clinical |
| 20A-79 | 20.00 | ND | 21.16 | *cstIII* | Clinical |

^a^Ct, Critical threshold. ^b^ND, not detected. ^c^Neg, no *cst* genes detected.

*Isolates with whole-genome sequences.
